# A catalyst for change: Developing a collaborative reflexive ethnographic approach to research with hospital doctors during the COVID-19 pandemic

**DOI:** 10.1177/20597991221137813

**Published:** 2022-11-29

**Authors:** Jennifer Creese, John-Paul Byrne, Rebecca Olson, Niamh Humphries

**Affiliations:** 1 Department of Population Health Sciences, University of Leicester, Leicester, UK; 2 Graduate School of Population Healthcare Management, Royal College of Surgeons University of Medicine and Health Sciences, Dublin, Ireland; 3 School of Social Science, The University of Queensland, Saint Lucia, QLD, Australia

**Keywords:** Qualitative research, methodologies, collaborative research, reflexive practice, remote research

## Abstract

The understanding of what ethnography looks like, and its purpose, is continuously evolving. COVID-19 posed a significant challenge to ethnographers, particularly those working in health-related research. Researchers have developed alternative forms of ethnography to overcome some of these challenges; we developed the Mobile Instant Messaging Ethnography (MIME) adaptation to ethnography in 2021 to overcome restrictions to our own research with hospital doctors. However, for ethnographic innovations to make a substantial contribution to methodology, they should not simply be borne of necessity, but of a dedicated drive to expand paradigms of research, to empower participant groups and to produce change – in local systems, in participant-collaborators and in researchers and the research process itself. In this paper, we reflect on our experiences using MIME, involving collaborative remote observation and reflection with 28 hospital doctors in Ireland from June to December 2021. After reviewing literature on ethnography in COVID-19 and general epistemological developments in ethnography, we detail the MIME approach and illustrate how MIME presents an evolution of the ethnographic approach, not only practically but in terms of its reflexive shift, its connected and co-creative foundations, and its ability to drive change in research approaches, participant life-worlds and real-world improvement.

## Introduction

The COVID-19 pandemic – in addition to unsettling the world along with health and societal systems – confounded the best laid research plans. New ethical paradigms, institutional policies and life pressures arose, challenging and often scuppering the work of scholars, ethnographers in particular (Fine and Abramson, 2020; Góralska, 2020; Lobe et al., 2020; Newman et al., 2021). At the same time, contemporary ethnographers have for many years been wrestling with the conceptualisation of ‘impact’ in their work, particularly under evolving funding frameworks. New ethical debates and dilemmas have arisen in the nexus between the imperative to effect real-world change – often dictated by funders and policymakers – and the ethics of honouring the contextualised human emotions, experiences, influences and challenges of the ethnographic participants directly impacted by both the research and subsequent changes (Bergerum et al., 2020; Naumann et al., 2020; Olson and Dadich, 2022).

Two driving forces – COVID-19 and the push for evidence-informed healthcare improvement – are catalysing change for researchers aiming to improve healthcare practices and systems. Conducting ethnographic research in this setting is complex. The ‘ethics of care’ (Askins and Blazek, 2017: 1089) of research is pulled between the need for evidence-based responses to the pandemic, the call for impactful research to underpin far-reaching healthcare improvement, the risks of working around a highly contagious virus, the need to amplify healthcare workers’ voices and the imposition of a greater load on workers (in health and academic settings) already stretched to the limit. Different ways of doing research for change – and of thinking about the way research changes all involved – evolve in this space. In this paper, we describe and reflect on our experiences of using a newly-developed Mobile Instant Messaging Ethnography, or MIME, method to remotely explore the working lives and experiences of hospital doctors in Ireland during the COVID-19 pandemic. We reflect on the way in which MIME, although an innovation born of COVID-19, is not merely a response to the need for change in ethnographic practice, but is also itself a catalyst for change itself: in methodological approach, in researchers’ conceptualisations of ethnography, in participants’ understandings of their own working lives, and potentially for healthcare improvement.

## Ethnographic approach: Beyond ‘being there’

In discussing ethnography as a methodology, there is substantial debate around the nature of what makes something ‘ethnography’; legitimacy in ethnography has long been built on the value of data gathered through physical presence and observation (Chambers, 2020). This particularly centres around the concept of ‘fieldwork’ and the ‘field’ of ethnographers’ work. A ‘field’, as defined within anthropological ethnography, is ‘the place where the distinctive work of “fieldwork” may be done, . . .[where a] culture or society lies waiting to be observed and written’ (Gupta and Ferguson, 1977: 2).In the anthropological tradition, this involves long-term immersion into the site being examined, participating in activity as much as possible across as many spaces of the site as can be accessed and drawing together a holistic view of multiple vantage points from across different groups and positions of those within the site.

However, such observation is not always possible or suitable for answering questions about lived experiences. Sometimes observation is not safe, or not permitted, as during COVID-19. In other cases, it may not be appropriate for analysing the everyday experiences of people whose cultural or social practices do not take place in physical spaces, such as online communities, or people whose cultural or social experiences take place physically in their own local spaces but are ruled by larger non-physical social institutions (Smith, 2005). In these cases, traditional physically-present participant observation fieldwork may not be the best way to observe and write about culture and society. Ethnographic methodologist Wolcott (2003: 113) goes so far as to say that ‘the *idea* of being there represents an idealised and romantic view of how fieldwork should be conducted’ – that is, that the paradigm of traditional in-person participant observation as the only true form of ethnography discounts the validity of undertaking other research approaches, not involving physical co-presence, with the epistemologies of ethnography in mind.

There are many well-established ethnographic approaches and methodologies, including institutional ethnography (Smith, 2005), digital ethnography (Varis, 2015) and nonlocal ethnography (Feldman, 2011), which do not require an on-site presence of any kind to still be considered ‘fieldwork’. Rather than being conceived of as sites, these approaches conceive of the ‘field’ in a Bourdieusian (Bourdieu, 1986) sense as ‘a social space. . . the context in which agents act and invest to be successful within a specific area. . . using various forms of capital’ (Olsson et al., 2019: 445). In the case of our work (as in Olsson, Kalén & Ponzer’s), the medical profession itself constitutes the ‘field’ as experienced by the agents acting and investing within it. Within this conceptualisation of a field, ethnographies also need not be multi-positional to give a rich and holistic view; many ethnographic works considered classic have focussed on the particular vantage points of small and specific groups within the context of their activities within a field of their specific social space and capital, particularly in healthcare (e.g. Becker et al., 1961).

Thus, mindful of the semantic meaning inherent within the terminology of ‘ethnography’ and its participant observation traditions, MIME joins a group of established *ethnographic approaches* that eschew physical participant observation yet maintain a commitment to the epistemological standpoints at the heart of ethnography. We draw our understandings of such an ethnographic approach from Borgstrom (2018), where work that is ethnographic has:

*a commitment to make sense of people’s lives from their points of view; engagement with the field, however constructed by the researcher and through the project and not limited to a geographical area. . . flexible in the kinds of engagement involved in research. . .seek to place issues within wider context. . .[and] analysis that invokes working through descriptions even if these do not always feature as ‘thick descriptions’ in the various outputs produced by research. (Borgstrom, 2018: 67–68)*

The core features of this articulation are emic perspective, engagement, flexibility, contextualisation and description-based analysis. These are the foundations on which MIME was developed: to respond flexibly to the requirements and restrictions of research during COVID-19, but more than this, to describe, engage with and analyse participants’ lived experience in a way that empowers and explores their own experiential and emotional spaces within the broader social context of their professional worlds.

## Collaboration, reflexivity and ‘impact’: New ethnographic ideals

The ethnographic method is always evolving. From the reflexive feminist turn of the 1970s to the development of digital ethnography in the 2000s, ‘as cultures and societies develop, so does ethnography, using its inherent adaptability and flexibility to try and find out “exactly what is going on”’ (Varis, 2015: 64). As social research has become more impact-driven and more closely connected with institutional or ‘industry’ partnerships, participatory research and implementation science, ethnography has been reconceptualised. Shifting from the traditional model of ethnographer as data-dissemination-change producer, ethnography has become a vehicle for participant-led inquiry and local change, narrowing the translational gap between the producers and users of knowledge (Rycroft-Malone et al., 2015). Collaborative methodologies of ethnography have emerged from this reconceptualisation. Collaborative ethnography involves participants in any, or all, of the multiple stages of research, including scoping and design, data collection, data analysis and research output and dissemination. Such an approach is important in quality improvement initiatives, particularly in healthcare, which have long focussed on outcomes for the implementing organisation and organisational logics, either neglecting participant patients and staff or casting them as ‘passive resisters’ of change (Gadolin and Andersson, 2017).

Alongside the collaborative work of building the corpus of ethnographic research data, these new methodologies have often sought to involve participants in the processes of reflexivity, as ‘an embodied process of making ethnographies accountable’ (Bieler et al., 2021: 78). Such reflexive processes are implicated in the conceptual understandings they draw out and knowledge they produce that in turn shape the institutional responses influencing participants’ lives. The overarching philosophy of collaborative reflexivity is a joint approach between researchers and participants to understanding and articulating the ‘structuring structures’ (Bourdieu, 1984: 170) of participants’ ‘lifeworlds’ (Kraus, 2015) which contextualise the data and shape conceptualisation of what is observed. In collaborative reflexivity, including approaches like video-reflexive ethnography (Iedema et al., 2013), participants move from research subject to epistemic partner, promoting their ways of knowing in processes of sense-making (Criado and Estalella, 2018) and drawing emic and etic positioning and understandings of data together (McNess et al., 2015). At the same time, the reflexivity of the researcher becomes less distanced and discipline-framed, and the resulting work can better negotiate the tightrope between applied and theoretical legitimacy (Bieler et al., 2021). Here, collaborative dialogue and partnership strengthen the ethnographer’s interpretative authority, and lead to enriched and empirical knowledge with which to drive improvement – for example, change that was sorely needed in responding to the COVID-19 pandemic.

## Ethnography and COVID-19: Lemonade from lemons?

COVID-19 has greatly impacted on the work of ethnographers. In many places, work-from-home and stay-at-home orders governed the early pandemic. Even in locations where these were eased, many institutions discouraged or even outright banned face-to-face data collection (Arya and Henn, 2021; Lobe et al., 2020). Ethnographers themselves wrestled with the ethics of conducting their research, even if institutional permissions allowed them to work: alongside a risk to their own health, they now presented a ‘vector of danger’ (Fine and Abramson, 2020: 165) and a burden on the people and institutions involved in the research process. Work-from-home mandates meant that research had to happen in the home, alongside lives that were increasingly strained across home-work-life, including home-schooling and care for ill and socially-isolating friends and family (Marhefka et al., 2020).

Yet, institutions, systems and scholars alike also realised the importance of gathering insights and lived experiences of the pandemic to inform policy and practice responses and prepare for the future (Crivello and Favara, 2021; Harris and Schlosser, 2021; Leslie et al., 2020; Richardson et al., 2021). In response, the COVID-19 pandemic has been a catalyst for research change, not only for the increasing use and importance of rapid (Richardson et al., 2021; Vindrola-Padros et al., 2020) and digital (Góralska, 2020) ethnographic methods, but also in the training of healthcare staff in ethnographic and observational principles and practices to gather insights from within the provision of care (Palinkas et al., 2022; Shand et al., 2021). Additionally, many scholars have made a general call for greater and lasting methodological innovation in ethnography, not just in the face of COVID-19 but as part of a more responsive participant- and policy-focussed approach to ethnography (Arya and Henn, 2021; Rahman et al., 2021). It is within the context of this call, and the need for a collaborative approach to understanding health system experiences, that we have developed our MIME approach.

## Methods

The MIME method was developed as part of an Irish Health Research Board-funded study of the working conditions and emigration of hospital doctors in Ireland. After previously surveying and interviewing Irish hospital doctors at home and abroad (Byrne et al., 2021; Creese et al., 2021; Humphries et al., 2019a, 2019b), an ethnographic approach was deemed necessary to build up an understanding of what day-to-day work and working conditions looked like for hospital doctors in Ireland, over a longer period.

However, it was important not only to document the day-to-day experiences and cumulative pressures of work in Irish hospitals, but also to understand the incremental effects these experiences and pressures had on doctors’ working lives and their physical, emotional and social wellbeing. Stress, burnout and vulnerability to ill health are common among doctors generally (Smith et al., 2017); but in the Irish context these phenomena are alarmingly prevalent. Over 80% of doctors report extreme stress, almost 30% report burnout and 70%–80% report working when ill or avoiding seeking health treatments due to work (Hayes et al., 2017, 2019). Burnout is problematic in itself, impacting doctors’ well-being and career, patient experiences, care outcomes, health system costs and workforce retention (Humphries et al., 2014; Montgomery et al., 2019; West et al., 2018). This last factor is particularly problematic in Ireland where the medical workforce is ‘in crisis’ with high rates of doctor emigration (Humphries et al., 2019a). It was crucial to understand how the cumulative pressures driving doctor emigration and weak retention built up over time, what their sources were and how hospital doctors experienced them in the context of their professional roles and identities.

A traditional field-based hospital ethnography was planned to study the pressures and experiences of working in Irish hospitals. The arrival of COVID-19 made these plans unworkable. A new research approach was needed to gain a similar understanding of hospital doctors’ lived experiences over time, within the confines of hospital visitor inaccessibility and public health guidance (i.e. work from home mandates for non-essential workers and travel restrictions). We therefore developed a remote research study, utilising common but secure smartphone communication applications to connect researchers and doctors on a regular basis, enabling us to communicate with them and discuss their experiences and feelings about work. We labelled this method Mobile Instant Messaging Ethnography (MIME; Humphries et al., 2022). We sought a mobile and agile method, available for participating doctors to connect with researchers at any time during or outside their workday as events, comments or answers to questions occurred. We also sought a method which allowed instant interactivity, allowing us to replicate virtually the ‘hanging out’ nature of ethnographic observation (Ito et al., 2010). Basing connection on mobile-phone devices was an obvious choice due to their ubiquitous use for communication and work operations in modern medicine (Nair et al., 2021). We also elected to utilise WhatsApp, an instant messaging application, which doctors frequently use for work related communication (Gould, 2017), to allow for any-time one-to-one messaging between doctors and researchers in a variety of formats. Finally, we built on the foundations of the Mobile Instant Messaging Interview, or MIMI, technique (Kaufmann and Peil, 2020), but furthered our methodological design so that – although not replicating a traditional field ethnography exactly – we undertook the research in accordance with key principles of the ethnographic paradigm (albeit also within the constraints of the pandemic), in an approach more aligned with ethnography than traditional interviewing.

The MIME project sought participation from hospital-based doctors, of any grade or nationality, working in hospitals in Ireland at the time of the study. Twenty-eight hospital doctors elected to participate. Each was assigned to one of three project team members (JC, JPB, NH) and, after receiving substantial participant information and giving informed consent to participate, conducted a preliminary video interview with their researcher via Zoom or WhatsApp, establishing their work and life context, or ‘field’ Across 12 weeks, each participant and their researcher participated in WhatsApp text conversations at least three times a week, consisting of researchers’ questions and participants’ answers and unprompted comments or responses, on varied themes or about various events in their ‘field’. After the 12 weeks, participant and researcher met again via Zoom or WhatsApp for a final debriefing interview, where a reflexive discussion was held about key themes and elements arising across their MIME messages and about the processes of reflecting and observing themselves.

We have reflected elsewhere on the design, operation and experience of conducting research using our novel MIME method from an operational point of view (Humphries et al., 2022) this paper does not revisit these methodological practicalities. Rather, we aim here to reflect instead on how MIME has been a catalyst for change: in our conceptualisations as researchers of what it means to research ethnographically, in our participants’ conceptualisations of their ‘fields’ and their place within them, and potentially to inform improvement within the Irish health system.

## Findings

Here we outline our experiences of conducting research using MIME. First, we describe how interactions fostered descriptive insight into the context: a sense of participants’ ‘fields’. Next, we explore how collaboration and connection, on a personal and emotional level, developed between researchers and participants and shaped the study. Finally, we consider how the research exercise generated change, not only from findings but from the process itself, for both participants and researchers and their approaches to their respective work. In accordance with ethical clearance and principles, quotes are presented below using pseudonyms. Except where explicitly indicated, all participant quotes are drawn from follow-up reflexive interviews conducted after the 12-week period of MIME WhatsApp interactions was completed.

### Building ‘fields’: Developing context through MIME

One major concern of switching from traditional participant-observation fieldwork to participant-mediated MIME was whether the sort of rich contextual ‘thick description’ of ethnography could be accessed if the researcher did not experience the full sensory and affective immersion of ‘being there’. Thick description can be described as records of events and environments that go beyond surface appearances to interpret ‘how [people] shape and trim their actions to fit their principles’, and vice versa’ (Bosk, 1999 quoted in Leslie et al., 2014: 100). It also incorporates multi-sensory ‘phenomenological and embodied elements of ethnographic knowing’ (Pink, 2013: 261) by making sense of the ethnographer’s own immersed, sensory and affective perspective. In the context of health service research, this is important as it draws in all the ways that professional logics, hierarchies, standards, norms, frameworks and sensory environments shape the way healthcare happens and what is asked of healthcare professionals’ working lives. There can be a concern, in remote ethnography, that without first-hand field research, thick description might not be possible (Postill, 2017).

However, when participants engaged in regular conversations over multiple weeks, they began piece-by-piece to sketch a rich contextual picture not only of their hospital department and what sorts of things they did in it, but who and what else was part of that picture, and what other parts of their lives and careers beyond the hospital also shaped their experiences. By connecting with participants via smartphone, labelled a ‘transportal home’ which is ‘best understood not just as a device through which we communicate, but also as a place within which we now live’ (Miller et al., 2021: 219), we were able to uncover ‘exactly what is going on’ (Varis, 2015: 64) in doctors’ lived experiences at work, home and beyond. This scope would not have been possible in a traditional site-based ethnography. Rather than the field being a site where the participating doctors were observed, instead participants sketched out and filled in their own arenas of lived experience revolving around their practice as a hospital doctor, with each participant bringing together their own conceptualised ‘field’. Participants described not only the things they did each day, but the broader professional, personal, institutional and systemic contexts in which they did them – MIME was a mutual reflective process in which researcher and participant helped each other identify the structuring structures of hospital doctors’ working lives. Although limited by an inability to convey the same sensory and embodied perspective of the researcher in contextualising the field as traditional in-person ethnography, MIME did have the capacity to draw on a deeper emotional experiential context to develop description of a ‘field’ that was not only experienced physically, but psychologically and emotionally and shape it with ‘explanatory and emotional richness’ (Ewing and Pankauskas, 2013: 128).

The following two transcript poems (Glesne, 1997), made up of non-sequential verbatim statements from MIME WhatsApp interactions with two specific participants, demonstrate how they sketch out their ‘fields’ through MIME:

*No major catastrophes but busy with back-to-back patients*

*There’s always more to be done.*

*Have been feeling unsupported by colleagues and CEO*

*IT issues still causing problems. No remote access to emails.*

*See end of summer coming and don’t feel refreshed and wondering what the winter will bring.*

*Doctors understand the challenges and obstacles in delivering an efficient, safe service*

*But voices not heard amongst management.*

*I don’t have time in work to do ‘resilience training’ . . . [or] ‘lunchtime yoga’.*

*Need . . . computers that work, charts pulled for clinic, letters typed on time, photocopiers with paper in them.*

*Chronic understaffing . . . has left people over stretched*

*Nobody has time to take leave and when they do the work will just be waiting for them on return.*

*Covid has tipped the work life balance more towards work.*

*How can I keep up this pace until retirement? (R20)*

*Inpatient service very busy.*

*Reg on leave. . .so things a bit tight. . .going to be disaster.*

*Your patience gets extremely thin at the end of a 72-hour shift,*

*Really blunt, you don’t realize how far gone you are.*

*No doctor’s office so every conversation takes place on the ward,*

*Everyone can hear.*

*No spare computers, nowhere to keep a coat.*

*I’m a two-hour drive from my life.*

*Ten years of your life or more, filled with stuff you have to endure.*

*I’m very tired now,*

*Buying takeaway chips and going to bed. (R22)*

The themes here help build a picture of participants’ day-to-day activities in the context of their broader organisational, systemic and influences and pressures – as ‘thick’ a description of work-as-done as possible. MIME responses via WhatsApp captured the tasks of the day as participants had done them – and often the ‘extreme work’ (Humphries et al., 2019b) this entailed – as well as a sense of their working lives, the physical and organisational work environment and the professional and system frameworks and cultures that governed them. The responses were especially valuable in an ethnographic sense as, coming directly from participants’ lived experience, they naturally took an emic perspective. Additionally, participants noted that the MIME process also elicited for them a particularly ‘thick’ sense of what they themselves did day-to-day and how their experience of work within their hospital might fit into a broader view of the health system, one which they did not often have the time or space to consider:

*Your questions then gave an opportunity to reflect on where we were at and we’re a small hospital but I suppose we could be very demonstrative of the greater situation. (R28)*

*It was weird to think about, kind of, the bigger picture, because you can get very caught up in the minutiae. [But] the bigger picture stuff is borne out of, you know, individual experiences. (R9)*

This ‘bigger picture stuff’ formed the context in which participants’ own ‘fields’ were situated, bringing a rich interconnectedness across the data. While each participant’s experiences at work were their own, with their own backgrounds and frameworks, cross-cutting themes enabled the creation of broader insights into what work in Irish hospitals is like. This third transcript poem provides a composite of quotes drawn from the MIME WhatsApp transcripts of several participants to illustrate this composite ‘field’:

*Let’s just get the patients sorted and out of here, so*

*Write prescriptions, write blood forms on triplicate carbon paper*

*Drop everything, run up to the department, perform the procedure*

*Skip lunch, stay late, do whatever it takes to get it done*

*Because that’s the right thing for the patient.*

*But*

*Do your day job, hit by a cyber-attack, COVID thrown in on top*

*I worked 10hrs on the floor and still huge waits to be seen*

*Treating patients on 20-year-old machines, stretched between clinic and wards*

*Not only asked to do your job, you’re asked to do everyone else’s too*

*Three people were crying at handover, we were just shattered.*

*And then*

*All the messing around with being paid or being paid back*

*Even things as basic as space for bags and lockers and coats*

*Little pointless barriers caused by how things are done*

*I don’t think management listen, or even care*

*I just feel we are heading for a cliff.*

### Collaboration: Connection, emotion and reflection

As Fine and Abramson (2020: 165) state, ‘if there is one profound truth about ethnography, it is that intimacy, and not distancing, is crucial’. A key challenge of remote ethnographic methods is the absence of ‘being there’ and ‘being then’ (Gray, 2016; Postill, 2017) to share, and be seen to share, in the lived experiences of participants’ ‘fields’. In evolving the ethnographic approach for MIME, facilitating connection between participant and researcher was key, to ensure we overcame the ‘not being there’ and fostered a strong sense of connection with participants across the digital distance. In particular, we recognised that ‘emotions have an essential role in building our field relations’ (Lo Bosco, 2021: 9), and were conscious that emotional tone might be more difficult to gauge and convey in text-based messaging, as other researchers have noted (Chen and Neo, 2019). However, as researchers, we observed that MIME enabled us to become more deeply emotionally connected and invested in participants’ lived experiences than online interviews had; ‘going home’ with participants via their smartphone, over the 3 months as we watched, listened and empathised about their work, we also shared jokes and holiday plans, cheered successes and commiserated over negative events, both professional and personal. We also experienced emotions related to what we witnessed through MIME messages, often manifesting in physical feelings, similar to those described by Gray (2016) in her remote ethnography: the gut-wrenches of dread over a participant’s bad day, and the heart-lightening of their exam success, a palpable exaltation or disappointment alongside them as we heard about their experiences (Humphries et al., 2022).

The participants, too, shared our feeling of connection, particularly the ability to talk through and about their emotional responses to work experiences, something which was difficult to do.

*I’m flat sharing with another [junior doctor]. . .you wouldn’t go into the same detail about the way that you’re thinking about [things], your hopes or the way that it’s affecting you. (R15)*

*You’d have mates who you’d chat to now and then, but it’s not [the same]. . .I’ve never, in a consistent way like that, reflected on what work is like. (R23)*

Some also felt like the process of MIME participation gave them time and space and permission to share their feelings about work which made room for genuine emotions, unlike in the original situation or event that they were reflecting on, where they would have normally compartmentalised them:

*It makes me so sad reading it [my messages]. . . I guess ‘cos you’re telling it to someone else you’re kind of formulating your own thoughts about it as well. (R5)*

*I felt like almost on the week that I was the most busy was the week that I actually typed the most – because I was the most angry. (R24)*

This ability to feel and share emotions about work itself, and about thinking about work, in a ‘space’ of psychological safety (Edmondson, 2018) was highly valued by participants, in terms of both their emotional wellbeing and their ability to voice concerns about their working conditions:

*It was actually kind of therapeutic to say ‘this is the annoying things’. And to kind of hand it over to somebody else [and] move on with my life. (R7)*

*It was kind of nice to get some of it off my chest. Nice to feel like there was possibly somebody out there listening, trying to get things to change. (R26)*

The choice of WhatsApp texts, rather than being limiting for emotive communication and rapport, were a key factor for facilitating connectedness. Participants noted that they felt a connectedness by virtue of the consistent long-term presence of the researcher in the WhatsApp space, one in which they regularly conducted work and engaged in work discourse, and one noted to be ‘a highly socialized form of interaction’ (Lobe et al., 2020: 1):

*I’d see it on my phone when I sat down in the evening, or once or twice even on my walk home. Just as I was thinking about stuff, you know? It was probably as close as we could get to you guys being here. (R2)*

*You wouldn’t text back sometimes when you’re just kind of fed up, and just wrecked. . .[but whenever] you’ve time, you could answer them. . .in the mornings when you’re on [the ward or] you get home. . .it was good. (R10)*

Through the medium of the mobile phone and WhatsApp instant messaging, the researcher was available to them whenever they wanted to interact with the research or reflect on work, both in terms of time and location, much more than they would have been in a traditional site-based ethnography, though this posed its own challenges for the researchers (Humphries et al., 2022). Allowing participants to engage when they wanted to, may have also levelled the power balance between researcher and participant which is often problematic in ethnographic research, particularly with more junior doctors (Råheim et al., 2016).

Key to building understanding of participants’ ‘fields’ through MIME was their understandings of and reflections on the context in which they worked, and combining this with researchers’ own understandings and reflections on hospital, system and professional structures and cultures. The cognitive exercise of reflection and reflexivity was not one that all participating doctors initially felt at ease doing. Some felt that the professional duties and logics of medicine did not encourage reflection or reflexivity:

*You don’t really take that much time to reflect. It’s difficult to build it in. . . .We’re not really taught that, you know? (R4)*

*You might rant to somebody, you know, or something like that, but you wouldn’t kind of stop and think quite as much. (R12)*

Others felt that, even if they had wanted to reflect, the ‘extreme work’ of medicine left them little opportunity to do so as they struggled simply to get through their workload and pressures:

*Do we get an opportunity to debrief? No, we don’t. (R8)*

*Previously, the only time when people are able to fill in things about their attitudes to work, is when they have time. And if you’re in a really bad place at work, you don’t have time for any of that. (R22)*

Others felt that, while describing their working conditions and the impact on their daily work was easy, having to think about and articulate the impact on them on a psychological and personal level was more difficult, and required a significant shift of mindset:

*Certainly the questions, especially the ones about well-being and how you feel, that’s not something I would normally think about during the day. (R1)*

*It’s unusual to be asked so many questions about work. And it’s not an easy topic. . . you wouldn’t really discuss those things. (R19)*

These reflections were not always easy for participants: two of the original twenty-eight formally withdrew from the study citing burnout, and another two dropped out informally. But, in the main, participants felt that MIME gave them an opportunity to convey their feelings about work into words in a reflexive way, perhaps more than would have been possible in an interview:

*I don’t know that I reflected that frequently [before]. . . So, actually, it was kind of beneficial to think about those things, you know, week on week. (R3)*

*There was no kind of time pressure to answer. I would see it, and then there would be things ruminating in my brain, and then I’d answer. . . I’d actually thought about it, I would be reflecting consciously. (R21)*

By facilitating participant doctors to ‘reflect consciously’, we were able to elicit and collect their reflexive understandings of the system in which they worked and the things they experienced in it. Combining this with our understandings, as researchers, of the Irish health system from our own and others’ previous research enabled us to situate participants’ experiences and reflections within a greater body of knowledge, but also both reinforced and challenged what we felt we already knew about doctors’ working lives in the Irish health system with doctors’ own interpretations. This distributed reflexivity not only gave researchers a stronger emic foundation from which to help push for participant-informed change, but empowered participants to draw from their enriched understandings of their roles and experiences, honed by affect and emotion, to create change for themselves, at work and in their own lives.

### Creating change

The goals of publicly-funded research align with policy and practice change and improvement (Olson and Dadich, 2022); this research project is no different. However, MIME facilitated change long before the formal policy-informing stage of the research project; change has been driven not just from the findings, but from the very research process itself. For both researchers and participants, taking part in the MIME study changed our understanding of what we do and why we do it, how it impacts on us and others, and how we might in turn improve what we do for the ultimate goal of health system improvement.

For researchers, MIME challenged and changed not only the way we conceive of ethnography, but the way we practice improvement research and who we engage to see improvement happen. Our ideas of what constitutes ‘ethnography’ have changed, with previous learning and experience from our various disciplines about what ethnography ‘looked like’ as a method. Instead, we learned through MIME to see ethnographic research not as a method to be used, but as an approach to be embraced: in Borgstrom’s (2018: 67, 68) words, ‘one of not just ‘being-in-the-world’ of our research site, but of being in particular kinds of ways in relation to the wider research endeavour. . .which can be lost when ethnography is reduced to method’. It has also seen us take on an approach grounded in a stronger feminist praxis, an ‘ethics of care. . . meeting the needs, and maintaining the worlds, of ourselves and others’ (Askins and Blazek, 2017: 1089), not only for participants but also for researchers. Regular debriefing sessions became part of our standard practice within the research team, and a similar final debriefing session was held one-to-one with each participant (Humphries et al., 2022), reflecting a change in our understandings of the ‘politics of care’ inherent within our research. We have also, in exploring new approaches to gathering data, learned and tried other methods for sharing data, such as the poetry featured in this paper. One innovation (MIME) has opened us up to considering other innovations in research. Finally, in conceiving of engagement and system change, we have re-visualised our approach to research dialogue. Despite not having direct site connections through hospital-based ethnography, we aim not only to influence system change but also to create frameworks for the implementation of change at a local level: following a capability approach (Clark et al., 2019), empowering the voices and efforts of participating doctors (and by extension all doctors) to pursue their own values and objectives to improve their working lives.

For participants, the process of being involved in MIME, and particularly in talking and thinking reflexively about the things they did and saw around them in their hospital workplaces day-to-day, also began to change the way they approached their working lives. In the short-term, receiving a MIME message from the research team gave participants an excuse to reflect on work-related thoughts, emotions and anxieties and on the health system and their place within it, and then return to work with a changed attitude:

*Sometimes, even on busy days it’s nice to respond. . .to get your head out of whatever zone you’re in for a little while, because sometimes it brings balance to a situation when you go back to it. (R11)*

*It was a good way for me to vent my frustrations. (R17)*

Longer-term, the process of reflection and reflexivity across the 12 weeks of the MIME study led participants to adopt these processes as habit, and make changes of their own to their approaches and practices around work:

*You forced me to think a little bit more in the abstract in what I’m doing and pull myself out of the day-to-day stuff, and really think about it and it, and maybe reframe what I’m doing in some ways (R6)*

*To actually think about why, why was it awful day, and how might it have been better. I think actually, if we did more of that, we might be able to fix more things. (R20)*

Initial findings from our MIME data have been used to convey to policy makers and the public the significance of improved working conditions for healthcare delivery (Joint Committee on Health, 2022). But at another level of impact, participants indicated that thinking reflexively about their ‘fields’ and experiences throughout the MIME process, and the conversations and questions arising from interactions with researchers, have made some of them begin to push for change in their respective workplaces and organisations. While they admitted that this would not be an easy task in terms of changing established organisational cultures, on a local level they could now identify workplace challenges and work towards changing them:

*I try and say, “Well, that wasn’t ideal. And how do we do that better?” There’s only a certain amount of things that I can actually meaningfully influence. [But] if I do things differently, then it will have a difference on the outcome. . .small local things. (R14)*

This ability to provide a framework for reflexively making small local changes – ‘doing that better’ – is the heart of collaborative ethnographic approaches: as Iedema et al. (2013: 68) state, ‘what is critical in all this, ultimately, is not the solution itself. . .but rather that practitioners have the ability to articulate. . .how their work should unfold’. While top-down policy innovations may come from practitioners and researchers’ examinations and sense-making of work practices and fields, the ‘proof’ of their success is in impacts that make localised on-the-ground improvements in conditions, even in seemingly small ways (Moore and Buchanan, 2013), which facilitate individuals and teams to improve and to be the best they can be.

## Discussion: MIME as an evolutionary approach

Debates over the evolution of the ethnographic approach have long been a part of the discourse of qualitative social research. This is no less true in the context of a pandemic. While mindful of the need to safeguard both people and research itself, some commentators have challenged assertions that innovative forms born of the COVID-19 pandemic can be considered interchangeable with or equivalent to field-based participant observation (Fine and Abramson, 2020). In reimagining the ethnographic approach through MIME, we have attempted to retain the ethnographic principles of ‘understanding informants’ life-worlds and their situated practices and lived local realities’ (Varis, 2015: 56). MIME may be considered as somewhat of an ‘ethnography by proxy’ (Plowman, 2017), which delegates the collection of fieldwork data to those who are the ‘subjects’ of the field and sends it back to scholars for analysis. However, in MIME, we did not seek to have hospital doctors become ‘ethnographers’, either in the sense of doing the work of sustained and systematic taking of in-depth field notes or of utilising their position in relation to some ‘other’ or subject of the research to filter our access. Instead of merely a transferral of the ethnographic method to a new remote arrangement, we sought to modify our data collection methodology, drawing on principles of an ethnographic approach, into a completely new form. In this, we look to the post-qualitative paradigm, which aims to ‘imagine and accomplish an inquiry that might produce different knowledge and produce knowledge differently’ (Lather, 2013: 635). Post-qualitative methodologies call for a re-evaluation of research away from methodological purity towards theories and practices that are ‘culturally responsive, ethical, and methodologically flexible’ (Koro-Ljungberg, 2015: xxii). This aligns well with the goals of MIME – to fit to the particular styles and pressures of participants’ work experiences, to practice an ethics of care for participants and their professional logics, and to respond to challenges in the field with flexibility. We see MIME thus as a reimagination of ethnographic approaches, a way to engage with the lived experiences and structures of participants’ working lives in a way that is still ‘ethnographic’, but which preferences their own logics and contexts, minimises invasion while maintaining interactivity, reduces power imbalances and works together to think through the meanings, implications and potentials of their professional life-worlds.

Additionally, while collaborative work in ethnography has become well-established practice, alongside other collaborative approaches like participatory action research, typical projects are discretely bounded; participant collaborators and researchers interact in scheduled meetings, and communicate through the course of their work days, towards their shared desired outputs in the form of policies, protocols and academic literature. However, the connected and intimate nature of MIME through the use of smartphones and WhatsApp completely removed such boundaries. The ethnographer whose ‘field’ was engaged through social media could be ‘suddenly thrown into fieldwork’ at any time, producing an experience equal parts ‘reassurance to fatigue and exhaustion’ (Lo Bosco, 2021: 12). While this was not without its challenges to the researchers on a personal level (Humphries et al., 2022), it builds on ideas of immersion in the field that are at the heart of the practice of ethnography; namely, that in undertaking immersive participant observation one is ‘always “on”, that is, playing the role of being the participant observer and never able to escape’ (Musante, 2015: 268). Our experience with MIME suggests that remote collaborative work might also be ‘always on’, despite not being there for observant participation, by virtue of the online connectedness between researchers and participants, with stronger, deeper relationships and potentials. We see MIME as thus an alternative approach to collaborative ethnography: one where connection and shared reflection is as ‘always on’ as participants’ and researchers’ lives and thoughts are, and one which answers the call by Arya and Henn (2021: 10) for ‘greater co-creation with participants to gather more authentic understandings of their lived experiences’ through mutually-guided collaboration and reflection.

Further to this, context in ethnographic research has long been considered ‘an interactional achievement’ (Varis, 2015: 57) built with a blend of researchers’ and participants’ unique frameworks. Practices like distributed reflexivity have furthered this by engaging different disciplinary thought styles together, to ensure that the high-level theoretical or ‘concept work’ of research is entwined with ‘awareness of self within teams, systems or organisations key to developing distributed intelligence and the potential for locally appropriate solutions’ (McHugh et al., 2020: 673). Yet existing models of ethnography are still tied to the concept of field-as-place and reflexivity as the epistemic standpoints researchers and participant-collaborators bring into that space, seeing analysis as a ‘generative process that arises from engagements with the respective field sites rather than from distance to it’ (Bieler et al., 2021: 91). MIME shifts beyond this understanding of interactionally-constructed context born of collaborative reflexivity by redefining the ‘field’, not as a place but as a ‘lifeworld’ of the professional work and identity of participants; not just their ‘material and immaterial circumstances’ of lived experience but the ‘subjective construction of reality, which he or she forms under the condition of his or her life circumstances’ (Kraus, 2015: 4), shaped by their professional logics and understandings. A similar critical and self-critical reflexive analysis can thus be co-constructed, which performs this same sort of conceptually challenging and change-making work to improve practices and experiences of care regardless of field-site proximity, as the engagement and ‘being there’ are in the lived experience rather than the physical field. However, as researchers with an established research-based knowledge of the basic operations of the field setting, this co-construction could be undertaken without first navigating a substantial knowledge gap; further application and reflection of the MIME method is needed to establish whether it might be equally feasible an approach in field settings completely unfamiliar to researchers.

More than building understanding, such an approach informs health system improvement. Initial summarised findings arising from our MIME have already been delivered and discussed with high-level stakeholders, just as previous project studies have engaged system-level policymakers, representative bodies and regulators in attempts to drive change. Yet as an exercise in collaborative ethnography and reflexivity, MIME is an approach which is ‘constantly push[ing] the conceptual boundaries of the participating disciplines and professions’ (Bieler et al., 2021: 77) by encouraging both researchers and participants to conceive differently of their own roles in the research and in the improvement of the system. Researchers could see the practitioner-policymaker gap they needed to bridge by sharing the words and experiences of participants; participants could look at their daily struggles through a framework of articulating their wider impacts, needs and change mechanisms. The resulting outcomes of our research utilising MIME are twin-focussed: research with the traditional impact of informing policymakers, and the in situ impact of heightening participants’ ‘critical consciousness’ (Freire, 1973) of their circumstances and their distress, which itself has the potential to provide a catalyst for change on a local, national, professional and personal level.

## Conclusion

As demographic, technological and climatic changes usher in a new era of infectious diseases (Baker et al., 2022), novel viruses and pandemics like COVID-19 are likely to become more common. In this setting, we will continue to see impacts on societies, and health systems in particular, and ethnographers will continue to struggle to balance the risks and pressures of ethnography with the need for crucial knowledge, from lived experience. In this context, researchers need to be responsive, flexible and intrepid in the way they work, while maintaining a robust research approach and ensuring an ethics of care for themselves and their participants. The MIME method and approach profiled here presents an opportunity for ethnographers seeking to engage with participants’ fields in a socially-distanced, longitudinal way that centres an emic view of their life and thoughts and can be considered appropriately ‘ethnographic’.

However, beyond the confines of the COVID-19 pandemic, MIME presents an approach that may be a real catalyst for change, in the way we might do ethnography for health service impact and improvement. In drawing on contexts, findings and meanings from the day-to-day experiences of participants in collaboration with them, MIME provides grounds for change on multiple levels. First, it challenges and empowers participants to reflect on their work-life context, and make their own changes, about their work and systems. Second, it challenges and empowers researchers to change the way they see themselves and their research as part of the approach to system improvement. Finally, and most importantly, by the amplification of frontline voices through collaborative reflexive research, it has the capacity to challenge systems to change, to better meet the needs of those who spend their lives making the system ‘work’.
